# The Evaluation of the Modified Wave Periodization Model Efficiency on the Example of Young Soccer Players' Sprint Tests

**DOI:** 10.5114/jhk/191699

**Published:** 2024-09-26

**Authors:** Marta Szymanek-Pilarczyk, Michał Jakub Nowak, Tomasz Góra, Łukasz Oleksy, Miłosz Drozd, Jacek Wąsik

**Affiliations:** 1 Faculty of Physical Culture Sciences, Jan Dlugosz University in Częstochowa, Czestochowa, Poland.; 2 Faculty of Physical Culture Sciences, Collegium Medicum im. dr. Władysława Biegańskiego, Jan Dlugosz University in Częstochowa, Częstochowa, Poland.; 3 Department of Physiotherapy, Faculty of Health Sciences, Jagiellonian University Medical College, Krakow, Poland.; 4 Department of Orthopedics, Traumatology and Hand Surgery, Faculty of Medicine, Wroclaw Medical University, Wroclaw, Poland.; 5 Institute of Sport Sciences, Jerzy Kukuczka Academy of Physical Education in Katowice, Katowice, Poland.

**Keywords:** soccer, youth athletes, speed, long term athlete development, tactical periodization

## Abstract

The research aimed to evaluate the modified model of wave periodization efficiency in running speed tests conducted among soccer players aged 12 to 16. Participants included prospective players of a leading Polish top league soccer club. The research was carried out from 2018 to 2022 in June (Testing A) and December (Testing B) of each year. The test involved 30-m straight line running with 5-, 10-, and 30-m split time measurements. For this purpose, electronic photocells were used (FITLIGHT, Canada). The six-month training intervention increased the athletes' speed as there was a considerable decrease in the running time over the distance of 5 m (F = 7.86; p < 0.001), 10 m (F = 73.99; p < 0.001) and 30 m (F = 127.55; p < 0.001). Analysis of running performance of young soccer players aged 12–16 showed a significant improvement in speed at distances of 5, 10 and 30 m, confirming training effectiveness based on the wave periodization model. The negative correlation between testing year and performance suggests the influence of biological development on players' speed. The COVID-19 pandemic has impacted training, which was reflected in reduced differences between test scores. Improving initial running technique can contribute to better match results, which emphasizes the need for an individual approach to the physical preparation of players.

## Introduction

In the pursuit of planning the development of young athletes worldwide, irrespective of their sport, coaches devise programs with the goal of enhancing athletic efficiency. This objective concentrates on maintaining consistent high-level sports performance while mitigating the risk of injuries (Kurt et al., 2023; Magoshi et al., 2023; Shan, 2023). In sports science, several established solutions exist for addressing these challenges (Blecharz et al., 2022; Lindblom et al., 2023; Mynarski et al., 2015; Robertson and Joyce, 2015; Rodríguez-Rosell et al., 2017). These solutions provide a framework for designing customized programs tailored to the specific requirements of an athlete, a team or a club, while also accounting for individual development needs (Freitas et al., 2023; McFadden et al., 2023). Soccer academies worldwide serve as a valuable source of inspiration for creating rules that enhance individual, group, and team competencies (Rongen et al., 2020; Towlson et al., 2023). The solutions developed by coaches, teams, and players to address these challenges carry a degree of risk due to the unique nature of the work environment. Additionally, competition intensity and the demands of the specific sport further increase the risk of failure (Cumming et al., 2018; Gdovin et al., 2023; Rongen et al., 2020).

Training programs based on "tactical periodization" (Mangan et al., 2022) are becoming increasingly popular among leading European teams. They aim to prepare players to meet matches' tactical tasks and physical demands, using a hybrid approach that focuses on holistic training without isolated elements, by the "train while playing" principle. The key to this system is the interaction of the methodological tenets and understanding the foundations of the game model through which players can be shaped in many areas (technique, fitness, and decision-making ability).

One of the most critical abilities in soccer is speed, which significantly influences match outcomes. Even a fraction of a second faster with the ball can determine whether a player scores the decisive goal or prevents it (Clark, 2018). Thus, speed is of paramount importance in modern soccer, which is becoming increasingly faster (Jovanovic, 2011). It has been established that players’ ability to achieve high linear velocity over short distances differentiates between their performance (Baumgart et al., 2018).

**Figure 1:**
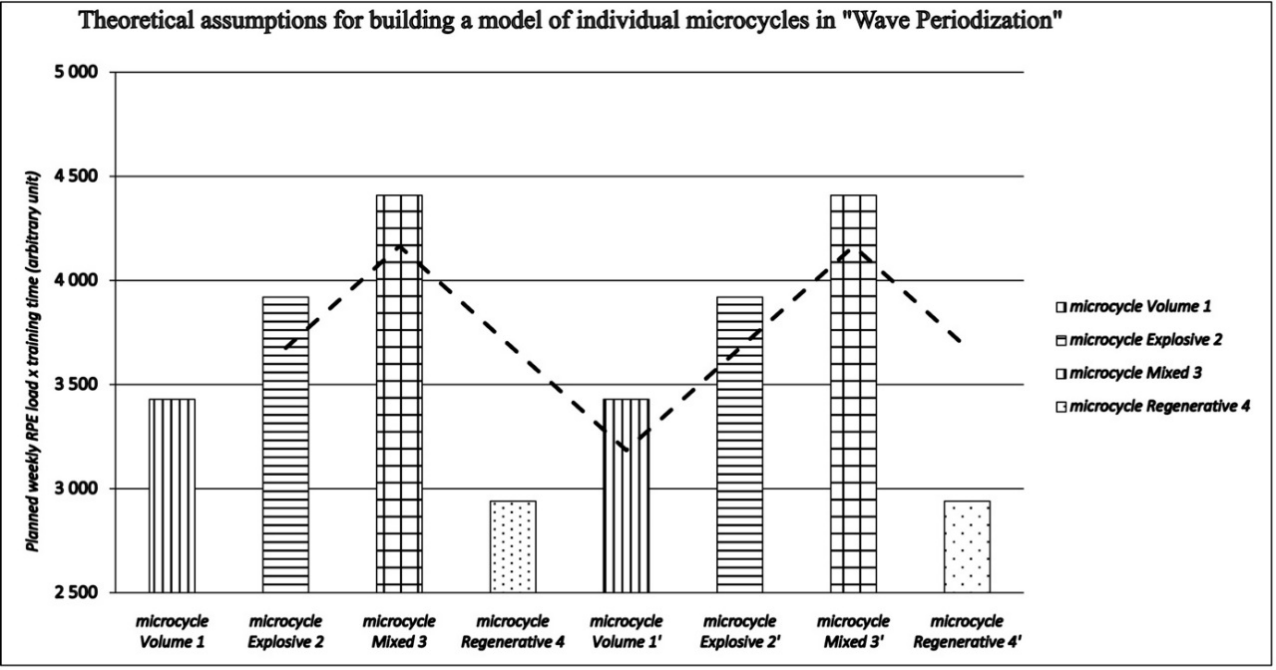
The figure shows the theoretical assumptions for the construction of individual microcycles in "Wave Periodization". The dashed trend line represents waves. The total training time per week is scheduled for 490 min. The height of the bar in the following weeks refers to the planned load of the athlete according to the following formula (planned intensity assessed by a subjective fatigue scale RPE on a scale from 1 to 10 x training volume (measured by the planned training time)).

The motor preparation model at the RKS Rakow Czestochowa Academy is based on a modified tactical periodization model, with the additional goal of fostering long-term development in children and youth (Szymanek-Pilarczyk et al., 2023). A training mesocycle, lasting 28 days, comprises four microcycles: volume (7 days), explosiveness (7 days), mixed (7 days), and regenerative (7 days). The volume microcycle takes place in large spaces, and the duration of individual games is long. Exercise intensity is low or moderate. The explosiveness microcycle is characterized by games in small spaces, shortened exercise duration, and an increased number of repetitions. Workouts are explosive, including various power-building exercises. The intensity of training measures is high or very high. In the mixed microcycle, training is conducted in large, medium, and small spaces, with the exercises' duration adjusted to the space's size. The intensity of the games ranges from moderate to high. The regeneration microcycle is characterized by training volume that is reduced each subsequent day. The main goal is to extend rest intervals to ensure recovery. Games occur in various spaces, but their intensity must be low. The described microcycles (system, terminology and characteristics) result from the need to implement tactical activities that are part of the training program of the RKS Raków Częstochowa Academy. The wave system of microcycles is implemented throughout the season, without preparatory, starting or de-training periods (Figure 1). The time structure of training remains consistent throughout the first and second rounds, with the exception of the regeneration microcycle, which aims to restore the mental and muscular systems. The microcycle's nature and motor tasks are aligned with tactical and technical considerations, including actions in specific game phases (attack, defense, transitions), intensity zones (low, medium, high), and sectors (side, center). Speed training is integrated into various types of games (small, medium, and large) and isolated training blocks that contribute to speed development. The main conditioning games are scheduled according to the match day (MD) + 3 days (MD+3) and the match day − 3 days (MD–3). On MD+3 training days, small-sided games (SSGs) and a speed development block (set of exercises) are implemented. The MD–3 involves games on the pitch of medium and large dimensions from 5 vs. 5 to 11 vs. 11 and a technique block (Chena et al., 2022). Speed activation is conducted on the day before the match (MD−1). Strength training also influences speed and is divided into upper body (MD+2) and lower body (MD+3) sessions. The game format depends on the number of players participating in the training session, and all games are closely connected to tactical objectives within specific microcycles.

**Figure 2:**
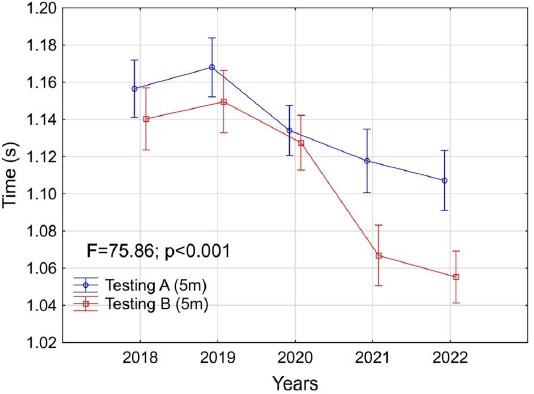
Changes in the average results achieved by players in the 5-m running test versus the year of the conducted assessment (from 2018 to 2022). The red line shows the results of testing A (in June), while the blue line shows the results of testing B (in December).

In recent years, there have been no long-term cross-sectional studies considering the latest trends in the development of youth soccer players. Therefore, this research aimed to assess the effectiveness of the modified wave periodization model in running speed tests conducted among players aged 12–16 at the RKS Rakow Soccer Academy (potential players of a leading Polish soccer club) in the years 2018–2022. We hypothesized that wave periodization would be an effective way to improve the speed variables of youth soccer players. We also examined the effects of the training intervention on running performance and the relationships between the selected results of soccer players who underwent testing.

## Methods

### Participants

The study sample comprised soccer players at the RKS Rakow Czestochowa Soccer Academy (Poland), aged 12 to 16 and with 4–8 years of training experience. Since all players training at the Academy took part in testing (with the exception of injured players who could not complete the tests), each year, approximately 100 players were evaluated (from 96 to 106 in particular years). Players belonged to the following age categories (mean ± standard deviation): U16: 19.5 ± 3.21; U15: 20.4 ± 1.52; U14: 20.2 ± 1.79; U13: 19.4 ± 0.89; U12: 19.4 ± 2.61. They trained six times a week, with four team training sessions, one formative training session and one match. Training included 90 min of gym training, 370 min of pitch training, and 45–90 min of game. The planned duration of training within a week (in min) for each player was approximately 490, regardless of the type of the macrocycle. All players adhered to the modified wave periodization model schedule. The same training plan covered each age group. Considering the participants’ performance level, they competed at the level of the central league in their age group or the first league in their district.

### Measures

Running speed was evaluated at a distance of 30 m. Electronic measuring equipment, i.e., FITLIGHT photocells (14845-6 Yonge Street, Suite 376. Aurora, Ontario, L4G 6H8 Canada), was used for time analysis. Measurements were made at distances of 5, 10 and 30 m from the starting point. The player’s foot was placed 20 cm in front of the first photocell line. Players began from a crouched start and they decided about the start time themselves. Each player performed two trials with 3-min rest intervals in between, and the results were recorded with accuracy of 0.001 s. All measurements took place under the same conditions, always in the same sports hall with a synthetic surface and temperature of 20°C. Players ran in sports shoes with indoor soles.

### Design and Procedures

#### Protocol

Before the tests, players performed a 12-min warm-up according to the RAMP protocol (Raise, Activate, Mobilize, Potentiate). The motor ability tests took place annually, with evaluations in June (Testing A) and December (Testing B) carried out after the competitive round in a specific microcycle with a standardized test protocol. The tests consistently occurred in the volume microcycle on MD+3 (the third day after the match). The day before evaluations, a recovery training session was conducted. All tests were performed in the same location, with uniform surface and temperature conditions (standardized). The tests took place at the same time, from 9:00 a.m. to 1:00 p.m. Before the tests all participants consumed a meal prepared by the clubs nutrition specialist (dietitian). All motor tests were carried out by experienced coaches (Masters of Science in Physical Education, ASCA Level I Trainers).

#### Training Program

Players adhered to the following schedule: MD+1 Day Off; MD+2 Recovery Training; MD+3 Small-Sided Games; MD−3 Medium-Sided/Large Games; MD–2 Day Off, MD–1 Pre-match Preparation. Training blocks that influenced speed were integrated at the beginning of each training session. The content of the speed block was closely aligned with the tactical requirements of the microcycle, encompassing different patterns, movements, and running distances associated with tactical actions in various phases of the game. The block shaping linear speed was administered on MD+3, and consisted of activation exercises and competitive sprints, with total duration of 15 min. The running technique block was implemented on MD–3 to teach or enhance the technique of specific running elements, with duration ranging from 12 to 15 min. Speed emphasis was applied on MD–1, including reaction time and acceleration elements. The strength training program was closely coordinated with speed sessions to ensure that strength exercises aligned with key movement patterns used during running.

#### Ethics

All participants were thoroughly informed about the study's content, objectives, possible risks, and benefits. The tasks and exercises performed were typical of their training, such as sprints and jumps. All participants held federation licenses, and parents had authorized their participation in the club's soccer activities at the start of each season. Interval training did not deviate from regular soccer training and did not introduce any additional risk. Additionally, all participants underwent medical examinations before the season, and the tests were conducted without injury or physical discomfort. This study aligned with the principles outlined in the Declaration of Helsinki. Approval was obtained from the bioethics committee at the District Medical Chamber in Krakow (approval number: 16/KBL/OIL/2016; approval date: 10 February 2016).

### Statistical Analysis

Sample size estimated using G*Power software (version 3.1.9.2; Kiel University, Kiel, Germany) (Faul et al., 2007) returned a minimum of 18 participants, for α = 0.05, effect size f = 0.75 and β = 0.95. The mean value and standard deviation were calculated for all indicators. The normality of the distribution was assessed using the Shapiro-Wilk test. Due to the non-normal distribution, differences between the compared groups were evaluated using Friedman ANOVA. Spearman's rank correlation coefficients were calculated for selected indicators. Statistical significance was assumed at p < 0.01. All calculations were performed using Statistica 13. The following scale was adopted to assess the strength of correlation: very weak: r < 0.10; weak: r 0.10 < 0.39; moderate: r 0.40 < 0.69; strong: r 0.70 < 0.89; very strong: r < 0.90 (Schober et al., 2018).

## Results

Table 1 presents the average results of assessments conducted in 2018–2022 for 12–16 age groups. As expected, the oldest players achieved the best test results (80% of cases; 24/30). The most deviations from this rule were observed in 2019 when 15-year-old players outperformed their oldest peers in three tests, and the most significant difference compared to the oldest group occurred in 2021 (December) when 14-year-old players obtained highest scores in the running test over a distance of 5 m.

**Table 1:** Summary of players’ results in running tests at distances of 5, 10 and 30 m, considering age groups, the year of testing and the month when testing was conducted (Testing A: June; Testing B: December).

Year of testing	Number of players (n)	Age	Testing A						Testing B					
			5 m		10 m		30 m		5 m		10 m		30 m	
			AVG	± SD	AVG	± SD	AVG	± SD	AVG	± SD	AVG	± SD	AVG	± SD
2022	24	**16**	**1.065**	0.078	**1.809**	0.072	**4.273**	0.104	**0.993**	0.045	**1.729**	0.057	**4.129**	0.111
	21	15	1.068	0.055	1.822	0.045	4.276	0.082	1.043	0.058	1.79	0.074	4.206	0.084
	18	14	1.097	0.073	1.866	0.074	4.451	0.163	1.054	0.061	1.823	0.069	4.289	0.110
	20	13	1.164	0.073	1.944	0.095	4.686	0.228	1.101	0.065	1.858	0.083	4.561	0.192
	16	12	1.162	0.064	1.980	0.051	4.875	0.125	1.109	0.053	1.923	0.073	4.729	0.178
2021	17	**16**	1.082	0.079	**1.777**	0.050	**4.172**	0.123	1.034	0.047	**1.743**	0.057	**4.146**	0.155
	21	**15**	**1.072**	0.062	1.79	0.061	4.211	0.134	1.024	0.043	1.754	0.056	4.209	0.133
	20	**14**	1.081	0.061	1.837	0.067	4.380	0.130	**1.017**	0.064	1.778	0.063	4.301	0.105
	18	13	1.161	0.073	1.925	0.077	4.642	0.194	1.133	0.081	1.916	0.099	4.603	0.202
	20	12	1.197	0.067	1.993	0.093	4.850	0.248	1.134	0.063	1.954	0.084	4.781	0.231
2020	22	**16**	**1.091**	0.052	**1.841**	0.056	**4.328**	0.145	**1.077**	0.062	1.812	0.073	**4.284**	0.139
	22	15	1.097	0.047	1.854	0.058	4.379	0.163	1.102	0.067	1.825	0.076	4.311	0.165
	20	14	1.155	0.067	1.933	0.091	4.567	0.241	1.112	0.056	1.877	0.078	4.514	0.172
	19	13	1.192	0.075	2.023	0.099	4.814	0.207	1.181	0.077	1.998	0.100	4.797	0.186
	23	12	1.145	0.056	1.971	0.075	4.868	0.156	1.171	0.066	1.981	0.075	4.864	0.188
2019	18	**16**	1.130	0.064	1.861	0.055	**4.361**	0.106	**1.100**	0.060	1.857	0.073	**4.342**	0.162
	20	**15**	**1.113**	0.059	**1.857**	0.057	4.418	0.193	1.112	0.088	**1.849**	0.081	4.362	0.159
	20	14	1.162	0.074	1.926	0.094	4.545	0.188	1.137	0.084	1.896	0.122	4.494	0.236
	20	13	1.201	0.066	1.973	0.097	4.686	0.252	1.195	0.074	2.027	0.229	4.658	0.281
	20	12	1.230	0.072	2.065	0.113	5.047	0.148	1.198	0.059	2.029	0.078	4.955	0.112
2018	17	**16**	**1.106**	0.052	1.882	0.055	**4.356**	0.122	**1.091**	0.067	**1.835**	0.060	**4.282**	0.118
	18	**15**	1.117	0.065	**1.860**	0.082	4.408	0.161	1.107	0.061	1.849	0.063	4.326	0.086
	23	14	1.159	0.077	1.924	0.083	4.540	0.178	1.113	0.066	1.876	0.064	4.412	0.159
	20	13	1.174	0.059	1.931	0.075	4.641	0.171	1.179	0.077	1.978	0.092	4.634	0.175
	18	12	1.222	0.07	2.046	0.085	4.815	0.227	1.212	0.078	2.042	0.085	4.841	0.293

AVG: average, ± SD: standard deviation, (n): number of players, values highlighted in yellow: best results in the 5-m sprint; green: best results in the 10-m sprint; red: best results in the 30-m sprint. Bold text: age category of players generating the best result

In subsequent years improvements were observed in 5-m sprint performance, with significant differences between particular tests (F = 7.86; *p* < 0.001). The most significant differences were observed in 2021 and 2022 (of approximately 0.06 s). The smallest difference occurred during the COVID-19 pandemic in 2020. There was a clear positive trend of improved results for each analyzed year.

**Figure 3:**
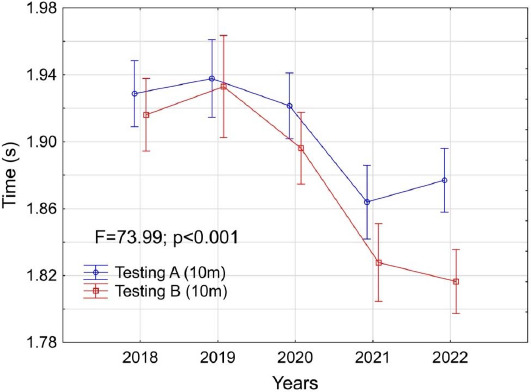
Changes in the average results achieved by players in the 10-m running test versus the year of the conducted assessment (from 2018 to 2022). The red line shows the results of testing A (in June), while the blue line shows the results of testing B (in December).

Figure 3 illustrates the average 10-m sprint test results over five years. Significant differences were observed between tests conducted in June and December (F = 73.99; *p* < 0.001) with most significant difference recorded in 2022 (of around 0.06 s). It should be noted that the size of this difference was related to the slower results compared to the previous year in tests conducted in June. The smallest difference between the average results and the 10-m sprint time was recorded in 2019.

present a very similar trend.

**Figure 4:**
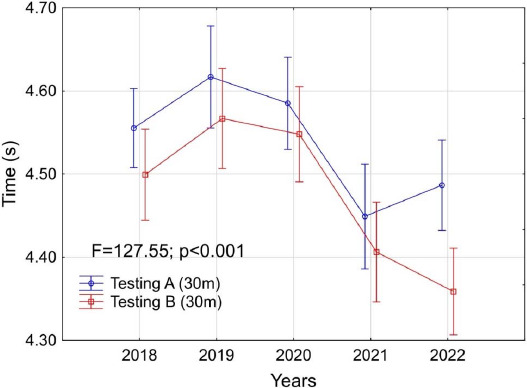
Changes in the average results achieved by players in the 30-m running test versus the year of the conducted assessment (from 2018 to 2022). The red line shows the results of testing A (in June), while the blue line shows the results of testing B (in December).

Figure 4 shows the average results for 30-m sprint performance. Statistical analysis showed significant differences between particular assessments (F = 127.55; *p* < 0.001). The largest differences between tests conducted in June and December were observed in 2022, while the smallest in 2020. Figures 3 and 4

Table 2 shows the correlation coefficients between the results of individual tests and the particular year of conducted evaluations. The year of testing showed a weak negative correlation with the results obtained at respective distances in both testing moments (June and December), which means that athletes of the same age ran slightly faster from year to year. Statistical analysis confirmed a high positive correlation between all conducted tests.

**Table 2:** The Spearman's correlation table of the selected indicators.

Variable		Testing A (June)			Testing B (December)		
		5 m	10 m	30 m	5 m	10 m	30 m
Year of testing		−0.24*	−0.23*	−0.16*	−0.41*	−0.35*	−0.23*
Testing A (June)	5 m		0.79*	0.67*	0.61*	0.62*	0.60*
	10 m			0.84*	0.65*	0.76*	0.77*
	30 m				0.62*	0.75*	0.89*
Testing B (December)	5 m					0.89*	0.74*
	10 m						0.87*

* p < 0.05

## Discussion

Our results confirm that training based on the wave periodization model significantly impacts running speed of youth (12–16-year-old) soccer players. Significant differences in the results of the 5-, 10- and 30-m sprint tests were recorded between 2018 and 2022. Considering every year included in the analysis, there was a notable difference between the tests conducted in June and December, indicating that interval training could lead to improved sprint performance. This improvement holds great significance in a match context, as high sprint speeds are crucial for professional soccer players' performance, especially under fatigue (Nicholson et al., 2021; Oliva-Lozano et al., 2023a, 2023b). In fact, sprints account for 83% of the time before scoring a goal (Faude et al., 2012). It is essential to emphasize non-linear sprints without ball possession in training, tailored to the specific tactical requirements of each player's position (Asian-Clemente et al., 2024; Beato and Drust, 2021).

1. Planned RPE volume microcycle at level 7 x training time; 2. Planned RPE explosive microcycle at level 8 x training time; 3. Planned mixed RPE microcycle at level 9 x planned training time; 4. Planned RPE regeneration microcycle at level 6 x training time.

The research shows an overall trend of improving running speed over five consecutive years, with the exception of 2019 when performance declined across all running trials. This decline was attributed to disruptions in the training process caused by the reconstruction of club training facilities, including the unavailability of a full-size sports field, changes in training schedules, and inconsistent access to gym facilities. Scientific sources support the influence of such factors on players’ development (Araújo et al., 2014). In 2020, the year of the COVID-19 pandemic, small differences were observed between testing in June and December, which can be attributed to the restrictions on regular training and the resulting irregularity, leading to significantly smaller progress.

Our results indicate a weak negative correlation between the particular year of conducted evaluations and the results of testing in June and December, indicating that players' biological development contributed to their speed. This conclusion aligns with existing research (Gundersen et al., 2022; Negra et al., 2022). The correlation coefficients clearly indicate that all speed test results are interrelated. For example, improvement in the 5-m sprint test conducted in June coincided with improved 10- and 30-m performance in December, reflecting the successful training process and evident enhancement of players running speeds across distances ranging from 5 to 30 m.

In conclusion, the wave periodization model proves to be an effective approach for long-term planning and speed development. The training regimen based on this model encompasses not only physical aspects, but also mental, physiological, and sociological dimensions (Mangan et al., 2022). This research provides an insightful analysis of the impact of a modified training strategy on developing players’ speed within a long-term program. It aligns with the observations of coaches and researchers and underscores the need for continuous players’ progress monitoring (Houtmeyers et al., 2021; Matusiński et al., 2021; Theodoropoulos et al., 2020). The authors aimed to address the gap regarding the long-term observation of youth soccer players by conducting an analysis of the impact of tactical periodization training conducted at a prominent sports club in Poland.

## Practical Implications

The research results provide valuable information for soccer coaches and strength and conditioning specialists that can be used to optimize soccer training. The data allow to identify key areas where training brings the most benefits, such as initial speed, acceleration, and speed-based endurance. Thanks to this, coaches can adapt training programs to players' individual needs, leading to more personalized and effective development of players and, as a result, better performance during matches. The idea of using the tactical periodization model (wave periodization) can undoubtedly be transferred to other team games in terms of motor preparation of players and development of motor abilities in relation to the requirements of a given position and the type of game.

## Research Limitations

The limitations of this work result from its general nature and long-term time horizon. The analysis does not include individual variables such as recovery and sleep quality, which are important for muscular and physiological adaptation (on the day of testing). Moreover, the study did not take into account the impact of the migration of players from other clubs who joined the Academy in older years. The integration of these players might introduce additional variability in performance, given their prior experiences and training, which might differ from the methods used in the Academy.

## Future Research Directions

Future research may focus on creating new percentile charts considering a range of speed, endurance and power. Such tools will enable a better understanding of norms and references for players at different levels of advancement, which may contribute to an even more individualized approach to training and sports development. These grids will also serve as a benchmark for assessing the progress and effectiveness of training programs.

## Conclusions

Training based on the wave periodization model significantly impacted the sprinting speed of youth soccer players aged 12 to 16. Statistical analysis showed significant differences in the results of sprint tests over distances of 5, 10 and 30 m conducted between 2018 and 2022. Furthermore, each year there was a noticeable difference between tests conducted in June and December. This indicates that training based on the wave model was effective and resulted in improved sprint performance. Additionally, a weak negative correlation was found between the particular testing year and results obtained in assessments completed in June and December, suggesting that players' biological development contributes to their speed. In the year of the COVID-19 pandemic, small differences between evaluations conducted in June and December can be attributed to limitations in regular training and the resulting irregularity, leading to significantly lower performance. The correlation coefficients indicate that the running speed test results are related.

Improvements in the 5-m test conducted in June correlated with improved 5-, 10- and 30-m results obtained in December, reflecting an effective training process and a marked improvement in the players' sprint speed over distances from 5 to 30 m. The observed changes in sprint test scores highlight specific areas where training resulted in the most significant improvements. A data-driven approach facilitates more personalized and effective players’ development, ultimately contributing to better match outcomes. Improved performance in short-distance sprints is associated with enhanced initial speed, allowing coaches to recognize that focusing on starting techniques can benefit the entire team. Analysis of the wave periodization pattern and identified correlations provide coaches with practical tools enabling a more precise and effective approach to the long-term physical preparation of players, which may translate into excellent results in team sports.
